# Hepatocellular carcinoma in identical twins in Chile: case report

**DOI:** 10.3332/ecancer.2016.708

**Published:** 2016-12-21

**Authors:** Christian Caglevic, Shirley Silva, Mauricio Mahave, Javiera Torres, Christian Rolfo, Jorge Gallardo, Paula Carrasco

**Affiliations:** 1 Unit of Investigational Cancer Drugs, Instituto Oncologico Fundación Arturo López Pérez, Santiago, Chile; 2 Radiation Oncology, University of Valparaíso, Valparaíso, Chile; 3 Anatomical Pathology, Pontificia Universidad Católica de Chile, Santiago, Chile; 4 Early Drug Development Unit—Phase 1 Studies, University Hospital of Antwerp, Antwerp, Belgium; 5 Oncólogo Médico Clínica Las Condes, Santiago, Chile; 6 Radiation Oncology, Clínica Alemana, Temuco, Chile; 7 Instituto Oncològico Fundación Arturo López Pérez, Rancagua 878, Providencia Santiago, Chile

**Keywords:** hepatocarcinoma (HCC), twins, brothers, relatives, genetics

## Abstract

Liver cancer is the second leading cause of cancer death worldwide, with hepatocellular carcinoma (HCC) being the most common type of primary malignant liver tumour, with a typically poor prognosis, growing incidence and a well-documented relationship with chronic inflammation factors of the liver tissue. Despite the fact that family medical history has been identified as a risk factor for the development of HCC, its significance in terms of etiopathogenesis and prognosis is not well documented. With a view to contributing to this discussion, we will report the clinical case of two identical twins with HCC, both diagnosed within a short period of time, by providing relevant clinical data, and relating this to other medical literature reports that could contribute to a deeper understanding of this illness.

## Introduction

Liver cancer is the second leading cause of cancer death worldwide (9.1% of the total, with 746,000 deaths in 2012). It has a high incidence which represents the fifth most common cancer in men (7.5% of the total) and the ninth in women (3.4%), with a poor prognosis (mortality/incidence: 0.95) and growing incidence [1]. Hepatocellular carcinoma (HCC) represents 70%–85% of all diagnosed primary liver cancers [2], its onset has been principally associated with chronic cirrhosis of the liver related to chronic infection by hepatitis B (this being responsible for 50%–80% of cases of HCC), besides chronic infection by hepatitis C virus, alcohol-related liver disease and non-alcoholic steatohepatitis, as well as other factors which include aflatoxins and other mycotoxins, diabetes, obesity and hereditary hemochromatosis [3–5].

Furthermore, a family medical history of primary liver cancer has become associated with HCC, and a synergistic effect with the infection of the hepatitis B virus [6, 7], with the vertical transmission of the virus being the probable mode of transmission in endemic regions [8].

Even though the relationship with family medical history has become associated as a risk factor for the development of HCC with reports of cases of HCC being reported in brothers [9, 10], for many authors, the family genetic contribution is determined by a susceptibility to carcinogenic environmental factors and many of the etiopathogenesis mechanisms involved in the illness are unknown [8]. Presented below is the HCC report on identical twins for the purpose of contributing towards the discussion of the role of genetic and environmental factors in the carcinogenesis of this tumour.

## Case reports

*Patient A:* 65-year-old male patient, from Temuco (Southern Chile), with a medical history of arterial hypertension, diabetes mellitus type 2 (DM 2), and cholecystectomy due to cholecystolithiasis. He had a consultation on 16 August 2013 because of an intense stabbing pain in the right hypochondrium. He was given a physical examination when admitted, and there was evidence of a hepatomegaly that was painful on palpation, without any other relevant findings.

A study with echotomography of the abdomen and pelvis was carried out (08-2013), finding a vascularised focal injury on the left liver lobe of 8.5 x 7 cm. A scan of the abdomen and pelvis (09-2013) revealed the presence of a solid substitute liver lesion with a neoplastic appearance in the left liver lobe of 10 x 7 cm, with central areas of necrosis. Alpha feto-protein (AFP) was ordered (09-2013) whose value was reported as greater than 350 ng/ml. The serology test was positive for VHB and VHC.

**Figure 1.**
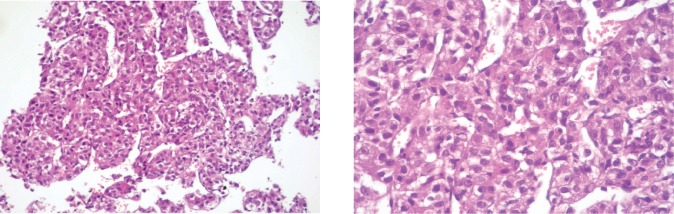
Microphotographs of the microscopic examination of material obtained by needle biopsy of a liver lesion with the following findings: residual type liver parenchyma with marked fibrosis and the apparent formation of bridges and lymphocytary infiltration at the interface level with areas of necrosis and the disappearance of the cells of the limiting plate. In other areas, a marked fibrosis can be observed with lymphocytary infiltration and proliferation of cell groups that are arranged in string and trabecular patterns formed by cells that have moderate nuclear and cellular irregularities, polymorphism and anisokaryosis with prominent nucleoli and a loss of the nucleo-cytoplasm relationship consistent with HCC.

A liver needle biopsy was performed on the tumour lesion (09-2013) whose microscopic examination showed the morphological appearance of HCC (Figure 1). The study was completed with immunohistochemistry, with the following results: CD10 (++) cytoplasmatic, hepatocyte (+++), AFP (++), which are consistent with a diagnosis of HCC. A molecular study of the biopsied material was not carried out due to a lack of access to this.

A PET CT (09-2013) was also performed which showed an ill-defined hypermetabolic liver lesion consistent with a history of HCC, 8.9 x 9 cm and SUV max, 11.7 and a hypodense hypermetabolic lesion on the left femoral neck. The biopsy is consistent with a HCC bone metastasis.

Treatment was started with chemoembolisation with the completion of three sessions that were carried out between October 2013 and February 2014. Later, surgery was performed to stabilise the lesion on the left femoral neck (02-2014) and palliative radiotherapy was given, 30 Gy in 10 sessions on this location, achieving a good analgesic response. Treatment was continued with zolendronic acid 4 mg IV every 28 days for 18 months and sorafenib 400 mg, twice a day since May 2014.

A few days after starting the treatment with sorafenib, the patient developed an increase in liver test levels, raised temperature (up to 38.5 °C) and increased blood pressure. The most significant findings of the laboratory tests were: (a) alkaline phosphatase, (b) 223 UI/L, (c) SGOT (AST) 414 UI/L, (d) SGPT (ALT) 670 UI/L, (e) bilirubinemia within the normal range, (f) lactate dehydrogenase 314 U/L and (g) elevated C-reactive protein (265 mg/L). A further abdominal ultrasound scan did not reveal any obstructive liver-biliary pathology. These symptoms were interpreted as drug-induced hepatitis, with the consequent suspension of the antineoplastic treatment, which was later restarted in June 2014 at a 50% dose with good tolerance. Once normal values in the liver tests were achieved, the dose of sorafenib was progressively increased without reaching the maximum recommended dose. Examinations of the thorax, abdomen and pelvis were carried out using a scanner (09-2014). These showed signs of progression in the primary lesion with portal vein thrombosis, signs of peritoneal carcinomatosis and regional lymph node enlargements, ascites and the previously detected secondary bone lesion in the right femur. October 2014 lab tests showed AFP:7865 ng/dl, plasma albumin 2,65 gr/dl, SGOT (AST) 78 U/L, SGPT (ALT) 81 U/L and FA 254 U/L with normal levels of total and direct bilirubin. A scan of the abdomen and pelvis revealed an increase in deep vein thrombosis and the appearance of signs of portal hypertension; therefore, a decision was made to suspend chemotherapy and keep the patient exclusively in palliative care. The patient died at the end of January 2015.

*Patient B:* Male patient, identical twin of patient A, 61 years old when diagnosed with HCC. He had a history of DM 2 and arterial hypertension; both pathologies were well controlled and being treated with drugs. He also had records of tobacco use and occasional alcohol consumption which were stopped in 1994.

In March 2010, he started showing symptoms of abdominal pain and a weight loss of 20 Kg in three months. The upper endoscopy showed the presence of esophageal varices and hypertensive gastritis. The abdominal ultrasound scan and the MRI of the abdomen showed the presence of tumour lesions of up to 11 cm in the dome of the liver, with satellite nodes consistent with HCC. Metastases were also observed in the right adrenal gland. The serology tests for VHB and VHC were negative. The AFP value was 2200 ng/dL in June 2010. The patient was referred to a centre of reference in Santiago de Chile in July 2010, where, furthermore, new images showed the presence of abdominal lymphadenopathy in the hepatic hilum, retroperitoneal and peritoneal lymph nodes. A biopsy was not taken and, as he had, an ECOG performance status of 1 treatment with Sorafenib was started. Unfortunately, 15 days after starting the treatment, the patient suffered a rapid clinical deterioration secondary to his aggressive underlying illness, leading to the suspension of the drug treatment and his death one month later.

## Discussion

HCC is one of the cancers where it has been possible to identify a large part of the risk factors with the support of strong evidence, and its principal association to chronic liver damage and cirrhosis of the liver [4]. In Chile, there are no official statistics of incidence and mortality caused by HCC; however, its relationship with cirrhosis of the liver (an illness with a high prevalence associated mainly to alcohol consumption) and chronic hepatitis caused by the hepatitis B- C viruses [11] is acknowledged.

Despite knowing the risk factors for this illness and recognising that the majority of patients with HCC have at least one identifiable risk factor, the development of HCC in the general population with such risk factors is considerably less; therefore, it is suspected that genetic variations could influence the risk of developing HCC in affected patients [12]. This fact, adding to the poor prognosis of the illness and scarcity of optimum treatments available in advanced stages, has encouraged multiple studies in recent years with the aim of understanding the complex pathogenesis, collaborating in an early investigation and developing new therapies that can improve the current prognosis of the illness. The development of HCC has been defined as a process of multiple steps, in which there is an accumulation of genetic and epigenetic alterations that will lead to the activation of oncogenes and the inactivation of tumour suppressor genes which will eventually lead to the initiation and progression of the cancer [13]. The identified mechanisms of hepatocellular carcinogenesis can include the following; malfunctions of the telomeres which lead to a chromosomal instability, mutations of the tumour suppressor gene p53 and the loss of its heterozygosity, mutations in the Rb and β-catenin gene, alterations in the Wnt signalling pathways, TGFβ and Ras pathways, changes in the expression of micro RNA, aberrant DNA methylation, histone modifications and the altered expression of long non-coding RNA (lncRNA), among others [13, 14].

Multiple reports of the family aggregation in HCC have been published in literature, principally associated with chronic infection by the virus hepatitis B virus [15–18]. However, genetic and molecular epidemiological investigations in these communities are scarce and although polymorphisms of some selected genes have been defined as risk factors for HCC, the results are confusing due to statistical and methodological limitations, and until now the effect of individual genes as risk factors for HCC could not be identified with sufficient evidence. The interaction of these genes with environmental factors is still being investigated, as well as their relationship with the phenotypic expression and prognosis of this illness [12, 19, 20].

In this context, the study of hepatocarcinogenesis in monozygotic twins represents an important contribution to the clarification of the HCC pathogenesis because of them sharing the same genetic code and environmental factors at an early age, making it possible to analyse epigenetic variations without the influence of confusing factors and separately identify the contributions of the environment and heritage [21]. In our review of medical literature, we can only find two previously reported cases of HCC in identical twins. The first report referred to twins with family intrahepatic cholestasis that died of HCC when aged ten [22]. In the second report, two 59-year-old twins simultaneously developed the illness, with chronic infection due to the hepatitis B virus, they were treated with doxorubicin but did not respond and they died a week apart; a genetic study was carried out on the tumours, finding a loss of heterozygosity of the retinoblastoma gene in the allele coding for XbaI RFLP, which has been interpreted by the authors as a firm suspect in the role of the hepatitis B virus in hepatocellular carcinogenesis, despite not having verified the integration of the DNA of the virus in the cellular DNA of these patients [23].

## Conclusion

In our case report, we found twins with findings of images consistent with chronic liver damage, with a diagnosis of HCC in adulthood, without relevant environmental records that present a rapid metastatic growth and a poor response to treatment. The diagnosis of HCC in these brothers within a short time frame is remarkable, coinciding with the previously discussed cases and with what has been reported in the medical literature on siblings that are not twins, in which cases, the time difference in diagnosis was no greater than five years [15]. It is not surprising that our patients were not observed to be chronic carriers of VHB or VHC, due to the situation in our country, which is considered to be a low endemic area for hepatitis B and C, with a risk for being a chronic carrier of less than 2% in a lifetime [24]. We can only identify DM 2 as an identifiable common risk factor in these cases of identical twin patients.

Regrettably, we could not carry out studies of genetics and polymorphisms in the tumours of our patients which could have shed light on possible factors of susceptibility for developing HCC. This would be interesting to address as there are no publications of this kind in our population, given that HCC is a growing health problem in our country.
